# Chronic granulomatous disease (CGD): an immunodeficiency diagnosed in pediatric ICU

**DOI:** 10.1186/1939-4551-8-S1-A88

**Published:** 2015-04-08

**Authors:** Ana Carolina Da Matta Ain, Erica Serrano Skamarakas, Adriana De Oliveira Mukai

**Affiliations:** 1 Universidade De Taubaté, Taubaté (SP), Brazil

## Background

This paper aims at showing a severe case of a patient with Chronic Granulomatous Disease (CGD), an immunodeficiency which is not so uncommon, affecting 1/250000 born alive, and which is due to phagocytic dysfunction. It is characterized by inability of phagocytic cells to produce hydrogen peroxide and other oxidants needed to eliminate microorganisms, in addition there is excessive accumulation of immune cells into aggregates, known as granulomas, at sites of infection or inflammation. The patient may experience fungal or bacterial infections affecting the skin, lungs (Aspergillus pneumonias), lymph nodes, liver (liver abscesses - 90% caused by Staphylococcus), bones (osteomyelitis), and granulomas which may lead to obstruction of gastrointestinal and urinary tracts.

## Methods

3-month old, female infant, admitted to Pediatric ICU with diarrhea, fever, abdominal pain and distention for 3 days. Umbilical stub fell off within 8 days. Previous history includes 2 episodes of suppurative otitis media. Physical examination at admission: tachypnea, abdominal distention, vestibulovaginal lesion, and extensive ulcerated anal lesion, with major hyperemia, sphincter laxity and edema. She further experienced septic shock, thus requiring fluid replacement, electrolyte imbalance and acid-base correction, blood transfusion, vasoactive drug, hydrocortisone, mechanical ventilation, and empirical antibiotic therapy (Oxacillin and Ceftriaxone). During the clinical course, there was the emergence of abscesses of right upper limb and flank and left lower limb (drained). She received human immunoglobulin, Piperacillin/Tazobactam, sulfamethoxazole-trimethropim, and fluconazole therapeutic dose. She was discharged from hospital on prophylactic antibiotic therapy.

## Results

She had leukopenia (2200 white blood cells/mm3). Abdomen X-Ray was performed due to worsening of abdominal distention, thus evidencing pneumoperitoneum requiring surgical intervention to remove the perforated segment of the colon. Rectal swab: Multisensitive *Pseudomonas aeruginosa*. Echocardiography: left ventricular dysfunction, ejection fraction: 43%. Chest X-ray evidenced thymus. Anti-HIV test – negative, normal immunoglobulins, normal lymphocytes, normal complements, Dihidrorhodamine (DHR): PMA-stimulated granulocyte population with abnormal response (low production of hydrogen peroxide).

## Conclusions

CGD is a life-threatening condition which must be suspected in patients developing severe infectious status.

